# NADPH oxidase 4 mediates TGF-β1/Smad signaling pathway induced acute kidney injury in hypoxia

**DOI:** 10.1371/journal.pone.0219483

**Published:** 2019-07-18

**Authors:** Sungkwon Cho, Seong-Lan Yu, Jaeku Kang, Bo Young Jeong, Hoi Young Lee, Chang Gyo Park, Young-Bin Yu, Dong-Chan Jin, Won-Min Hwang, Sung-Ro Yun, Ho Seung Song, Moon Hyang Park, Se-Hee Yoon

**Affiliations:** 1 Division of Nephrology and Department of Internal Medicine, Myunggok Medical Research Institute, College of Medicine, Konyang University, Daejeon, Republic of Korea; 2 Myunggok Medical Research Institute, College of Medicine, Konyang University, Daejeon, Republic of Korea; 3 Department of Pharmacology, College of Medicine, Konyang University, Daejeon, Republic of Korea; 4 Department of Biomedical Laboratory Science, College of Medical Science, Konyang University, Daejeon, Republic of Korea; 5 Department of Internal Medicine, The Catholic University of Korea, Seoul, Korea; 6 Department of Pathology, College of Medicine, Konyang University, Daejeon, Republic of Korea; Maastricht University, NETHERLANDS

## Abstract

Hypoxia is an important cause of acute kidney injury (AKI) in various conditions because kidneys are one of the most susceptible organs to hypoxia. In this study, we investigated whether nicotinamide adenine dinucleotide 3-phosphate (NADPH) oxidase 4 (Nox4) plays a role in hypoxia induced AKI in a cellular and animal model. Expression of Nox4 in cultured human renal proximal tubular epithelial cells (HK-2) was significantly increased by hypoxic stimulation. TGF-β1 was endogenously secreted by hypoxic HK-2 cells. SB4315432 (a TGF-β1 receptor I inhibitor) significantly inhibited Nox4 expression in HK-2 cells through the Smad-dependent cell signaling pathway. Silencing of Nox4 using *Nox4* siRNA and pharmacologic inhibition with GKT137831 (a specific Nox1/4 inhibitor) reduced the production of ROS and attenuated the apoptotic pathway. In addition, knockdown of Nox4 increased cell survival in hypoxic HK-2 cells and pretreatment with GKT137831 reproduce these results. This study demonstrates that hypoxia induces HK-2 cell apoptosis through a signaling pathway involving TGF-β1 via Smad pathway induction of Nox4-dependent ROS generation. In an ischemia/reperfusion rat model, pretreatment of GKT137831 attenuated ischemia/reperfusion induced acute kidney injury as indicated by preserved kidney function, attenuated renal structural damage and reduced apoptotic cells. Therapies targeting Nox4 may be effective against hypoxia-induced AKI.

## Introduction

Acute kidney injury (AKI) is defined as “an abrupt (within 48 hours) reduction in kidney function” [1]. The incidence of AKI gradually increases, reaching 8–16% of hospitalized patients [2]. AKI can progress to chronic kidney disease (CKD) and increase in-hospital mortality four-fold [2]. Despite the clinical importance of AKI, there is no absolute prevention or treatment has yet been found. Among the many causes of AKI, ischemic or non-ischemic kidney tissue hypoxia is the leading cause of AKI. Hypoxia not only leads to energy shortages in tissues, but also induces changes in intracellular signaling systems and gene expression. Kidney tubular epithelial cells are more vulnerable to hypoxia because these are less energy producing in anaerobic condition than other cells and consume more oxygen to maintain active transtubular absorption and excretion [3,4]. Various mechanisms have been introduced as mediators of the AKI caused by hypoxia, including calcium overload, endoplasmic reticulum stress, complement system activation, and reactive oxygen species (ROS) [5–9]. ROS play an important role in maintaining a normal intracellular signaling system in a stable state under normal conditions but the amount of ROS increases markedly in the pathologic state, acting as a crucial cause of AKI. Excessive production of ROS promotes hypoxia induced AKI by affecting the function of cellular DNA, proteins, and lipids [10,11]. Among various sources of ROS, nicotinamide adenine dinucleotide 3-phosphate (NADPH) oxidase (Nox) is the major intracellular non- mitochondrial ROS source. Because among seven Nox families, Nox4 is the most abundant in the human kidney, changes of Nox4 expression in hypoxia are predicted to affect the progression of AKI by altering the intracellular ROS level in the kidney. But few studies have investigated the role of Nox4 in hypoxia induced AKI [12–14].

Based on these observations, we investigated the role of Nox4 and the benefits of Nox4 inhibition in hypoxia induced AKI.

## Materials and methods

### Cells and reagents

HK-2 cells (a human renal proximal tubular epithelial cell line) were obtained from the Korean Cell Line Bank (KCLB, Seoul, South Korea). HK-2 cells were grown in RPMI-1640 medium with 10% fetal bovine serum and 1% penicillin/streptomycin. Cells were incubated in humidified, 5% CO_2_ –incubator at 37°C. CoCl_2_ (Sigma, St. Louis, MO) was used to induce hypoxia in the HK-2 cells at different concentrations (0, 100, 300, 600, and 900 μM). SB431542 was purchased from R&D Systems (Minneapolis, MN). The selective Nox1/4 inhibitor GKT137831 [2-(2-chlorophenyl)-4-methyl-5-(pyridin-2-ylmethyl)-1H-pyrazolo[4, 3-c]pyridine-3,6(2H, 5H)–dione] was thankfully supplied by Genkyotex (Chemin des Aulx, Plan-les-Ouates, Switzerland).

### Cell viability

Cell viability was measured with the 3-(4,5-dimethylthiazole-2-yl)-2,5-diphenyltetrazolium bromide (MTT) assay as reported previously [15–17]. Briefly, after hypoxia exposure, HK-2 cells were cultured in a 24 well plate. After 24 h incubation 5 mg/ml MTT solution (Sigma) was added to the wells and we incubated the cells for another 4 h. The supernatant was then removed and 1 ml of dimethyl sulfoxide (DMSO) was added to each well. Immediately after purple formazan crystals formed and dissolved, the solution was collected and pipetted into a 96-well plate. The optical density was measured at 590 nm (VICTOR X3; PerkinElmer, Waltham, MA, USA).

### Hypoxic induction with CoCl_2_

Here, CoCl_2_ was used to create a hypoxic condition [18]. CoCl_2_ can induce Hypoxia-inducible factor 1 α (HIF-1α) expression and rapidly and inexpensively induces hypoxic states in cultured cells in a dose-dependent manner [19,20]. HK-2 cells were treated with CoCl_2_ at different concentrations (0, 100, 300, 600, and 900 μM). Cell survival was reduced in a dose-dependent manner for 24 h (S1A Fig). Because approximately 70% of cells survived after treatment with 300 μM of CoCl_2_ for 24 h, we choose this dose for further examinations. HIF-1α levels increased dramatically in CoCl_2_ treated HK-2 cells (S1B Fig).

### Quantitative real-time polymerase chain reaction (RT-PCR)

Total RNA was extracted from HK-2 cells using Trizol (Invitrogen, Carlsbad, CA), and 1 μg of RNA was used for cDNA synthesis according to the manufacturer’s protocol and as described previously [15,16,21]. The assay used the following primer sets: *Nox-4*, 5’-GGCTGGAGGCATTGGAGTAA-3’ (forward) and 5’-CCAGTCATCCAACAGGGTGTT-3’ (reverse); *β-actin*, 5’-TCAAGATCATTGCTCCTCCTG-3’ (forward) and 5’-CTGCTTGCTGATCCACATCTG-3’ (reverse). Data were normalized to *β-actin* as an endogenous control. Relative expression difference were calculated by using the 2^-(ΔΔCt)^ method.

### Immunoblotting

HK-2 cells exposed to hypoxia for 24 h were washed twice with ice-cold phosphate-buffered saline (PBS) and lysed in a radioimmunoprecipitation assay (RIPA) buffer on ice. Whole cell lysates (50 μg) were subjected to 8% sodium dodecyl sulfate—polyacrylamide gel electrophoresis (SDS-PAGE) and separated proteins were transferred to polyvinylidene difluoride (PVDF) membrane. The membrane were blocked with 5% nonfat dried milk for 2 h at room temperature and incubated overnight with 0.2 μg/ml of primary antibody in PBS (pH 7.2) at 4°C. After two washes, the membrane were incubated with secondary antibodies (horseradish peroxidase-conjugated antibodies) for 2 h at room temperature. Signals were visualized using enhanced chemiluminescence (Thermo Fisher Scientific Inc., Rockford, IL) using Image Quant 400 (GE Healthcare, Buckinghamshire, UK). Following antibodies were used for this study. Nox4 antibody was from Abcam (Cambridge, MA). Antibodies for pp38 and p- c-Jun N-terminal kinase (JNK) were obtained from Cell signaling Technology (Denver, MA, USA). Anti-p38, JNK, p- extracellular signal-regulated kinase (ERK), ERK, Glyceraldehyde-3-phosphate dehydrogenase (GAPDH) were purchased from Santa Cruz Biotechnology (Santa Cruz, CA, USA).

### Caspase 3/7 activity assays

Hypoxia exposed HK-2 cells were incubated with Caspase-Glo 3/7 substrate reagent (Promega) at 37°C for 30 min. The samples were transferred to white-walled plates, and the luminescence signal was measured using a Lumat LB953 luminometer (EG&G Berthhold).

### Reactive oxygen species (ROS) measurements

Hydrogen peroxide (H_2_O_2_, end product of Nox4) was measured with Amplex red assays using Amplex red hydrogen peroxide/peroxidase assay kits (Invitrogen) according to manufacturer recommendations as described in author’s previous studies [15, 16]. Briefly reactions containing 50 μM Amplex Red reagents and 0.1 U/mL catalase in 50 mM sodium phosphate buffer (pH 7.4) were prepared in a darkroom under a red light. White enzyme-linked immunosorbent assay (ELISA) plates containing the samples (100 μL/well) were kept in the dark for 30 or 60 min. Fluorescence (excitation, 535 nm; emission, 595 nm) was measured on an HTS Multi-Label Reader (Perkin Elmer).

For the measurement of intracellular superoxide, the oxidative fluorescent dye dihydroethidium (DHE) was obtained from Thermo Fisher Scientific Inc. Cells were grown on glass slides in 12-well plates. Cells were fixed with cold methanol for 10 min and permeabilization for 30 min with 1% bovine serum albumin, cellular immunofluorescence was evaluated by staining with DHE for 40 min. The nuclei were counterstained with 4′,6-diamidino-2-phenylindole (DAPI; Molecular Probes, Carlsbad, CA, USA), and the cells were then washed with ice-cold PBS three times for 5 min and examined by confocal microscopy as previously described (LSM710; Carl Zeiss, Jena, Germany) [15,16].

For the measurement of intracellular hydrogen peroxide, Cells were loaded with 10 μg/mL of 2′,7′-dichlorodihydrofluorescein diacetate (DCF-DA; Sigma-Aldrich) and incubated in 5% CO_2_ at 37°C for 30 minutes. This result was confirmed by measuring cellular peroxide levels by 10 uM dihydrorhodamine (DHR) 123 (Sigma- Aldrich). Mitochondrial superoxide production was measured with 5uM MitoSOX red (Invitrogen). Fluorescence of DCF, DHR 123 and MitoSOX Red were measured using an HTS Multi-Label Reader (Perkin Elmer) at excitation and emission wavelengths of 495 / 535 nm, 480 / 530 nm and 510 / 580 nm respectively.

### Quantification of transforming growth factor-β levels

HK-2 cells were incubated in the hypoxia chamber (APM-30D, Astec., Tokyo) for 48 h. Transforming growth factor (TGF)-β1 was measured by an ELISA kit for human TGF-β1 (R&D Systems Inc.) in the culture media at different times according to the manufacturer’s instructions. After the sampling steps were completed, the optical density of each well was measured at 450 nm (VICTOR X3; PerkinElmer, Waltham, MA, USA).

### Animal models

All procedure in this study complied with the regulations of the Institutional Animal and Use Committee of the Konyang University. The animal care protocol for the experiments performed in our study was approved by the Institutional Animal Care and Use Committee of Konyang University. Experiments were carried out using twenty Sprague-Dawley female rats, 7 weeks old and weighing 180-200g. The rats were maintained in cages on a 12 h light: dark cycle at 22°C and received water ad libitum and food. After 1 week acclimation period, rats were randomized into four groups and placed into separate cages: Group 1(control, normal saline pretreatment plus sham operation; n = 5), Group 2 (GKT137831 pretreatment plus sham operation; n = 5), Group 3 [normal saline pretreatment plus ischemia/reperfusion (I/R); n = 5] and Group 4 (GKT137831 pretreatment plus I/R; n = 5) (S2 Fig). Renal I/R injury progressed as previously described [22]. Briefly, two incisions were placed on both flank area and the arteries and veins were clipped with a clip for 45 minutes and then reperfused for 24 hours. Sham operation was performed in the same way as renal I/R operation, except for arterial and vein ligation. Group 1 and Group 3 were given 5ml/kg of normal saline by gavage daily for 5 days while Group 2 and Group 4 received 10mg/kg (5ml/kg) of GKT137831.

### Biochemical and histological analysis

The blood samples were collected for biochemical analysis and kidney tissues were harvested for histological examination from separate group of rats after 24 hour of ischemic injury under anesthesia with ketamine/xylazine (0.5 ml of 100 mg/ml ketamine combined with 0.05ml of 20 mg/ml xylazine) at a dosage of 0.55ml/100g body weight. Serum blood urea nitrogen (BUN) and creatinine concentrations were measured using a Fufi Dri Chem 3500 (FUJIFILM, Tokyo, Japan). Kidney tissues were dehydrated with ethanol and fixed in 10% phosphate buffered formalin. After embedding in paraffin, it was cut into 4 um thickness and then stained with hematoxylin-eosin (H&E) or periodic acid-Shiff staining (PAS) to observe the structural changes. In order to examine apoptosis of kidney cells, we performed a Terminal deoxynucleotidyl transferase dUTP nick-end labelling (TUNEL) assay according to manufacturer’s instructions as previously described (HRP kit DBA; Apotag, Milan, Italy). [15,16].

### Statistical analysis

All graphed data are presented as the means ± standard deviation. The results were analyzed using analysis of variance (ANOVA) or a Student’s *t*-test. P-values of <0.05 and <0.01 were considered to indicate statistically significant and highly statistically significant differences, respectively.

## Results

### Hypoxia induces Nox4 expression

**Fig 1:**
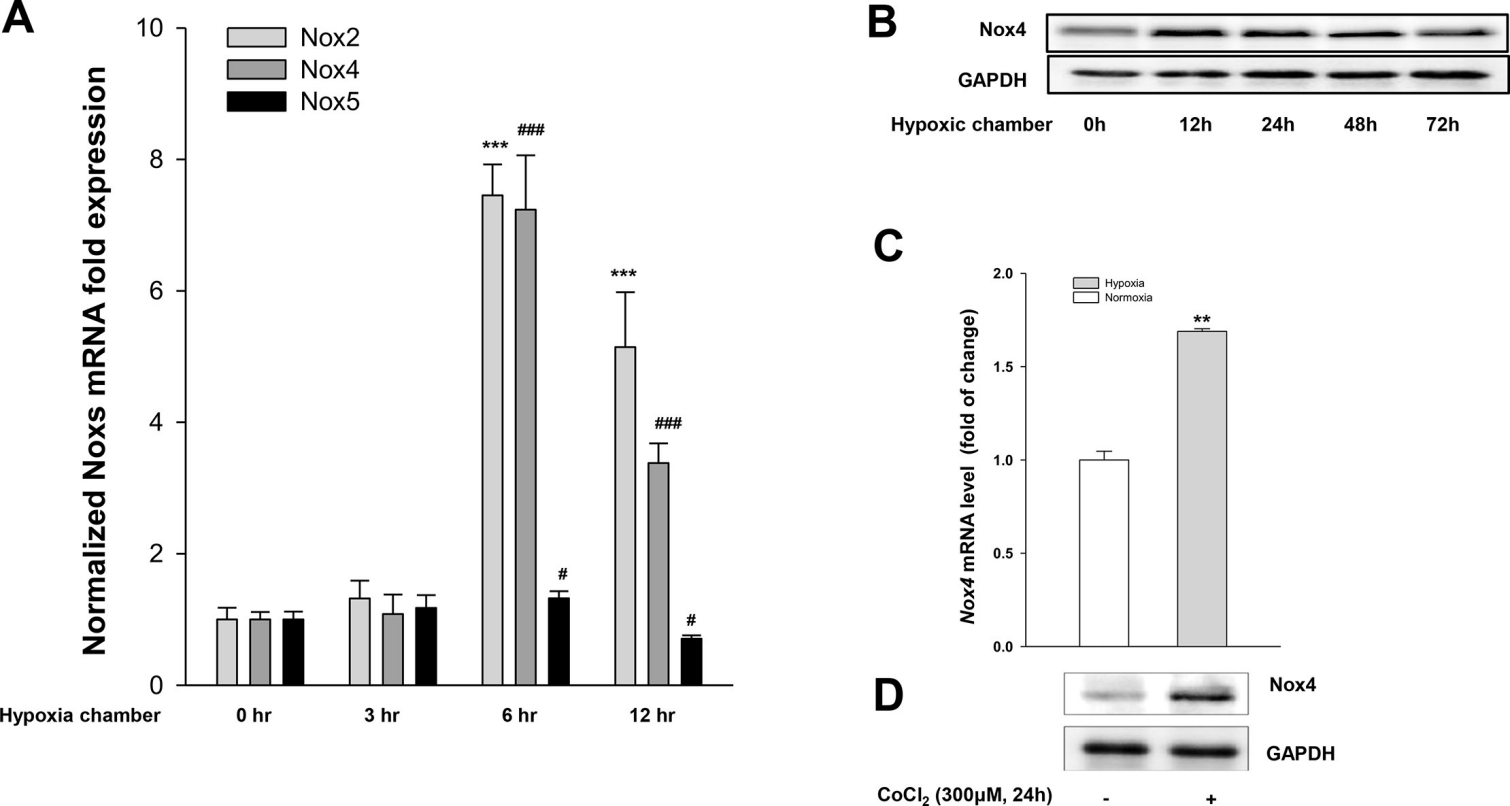
Hypoxia induces Nox4 expression. HK-2 cells were exposed to hypoxia using hypoxia chamber. (A) Quantitative real time PCR for Nox homologues (*Nox2*, *Nox4* and *Nox5*) at 0, 3, 6, and 12 h after hypoxia exposure. (B) Nox4 protein levels by western blotting at 0, 12, 24, 48 and 72 h after hypoxia exposure. HK-2 cells were exposed to CoCl_2_ for 24 h and analyzed at 24 h; (C) *Nox4* expression after CoCl_2_ exposure by quantitative real-time PCR; (D) Nox4 protein levels by western blotting. Data represent means ± SD; ***p < 0.001 vs. control and ^#^*p* < 0.05, ^###^*p* < 0.001 vs. hypoxia alone.

We explored the use of the HK-2 cell line as an *in vitro* model of hypoxia-induced kidney injury. We first investigated the effect of hypoxia on Nox homologues (Nox1, Nox2, Nox3, Nox4, Nox5) mRNA expression in HK-2 cells. Expression of mRNA was measured by real time-PCR after 0, 3, 6 and 12 h exposure to 1% oxygen, 5% CO_2_ and 94% nitrogen using the hypoxia chamber (APM-30D, Astec., Tokyo). As shown in Fig 1A, *Nox2* and *Nox4* mRNA expression were significantly increased after exposure 3 h of hypoxia. Expression of *Nox5* mRNA also increased after 6 h of exposure to hypoxia but was significantly less than that of *Nox2* and *Nox4*. Little *Nox1* and *Nox3* mRNA expression was detected in HK-2 cells.

We further examined the effect of hypoxia on Nox4 protein levels in cultured HK-2 cells for 72 h. Nox4 protein levels peaked 12 h after hypoxia exposure and maintained high for 72 h (Fig 1B). We also examined the effects of CoCl_2_ (300 μM) on Nox4 protein and mRNA transcript levels in cultured HK-2 cells. We found that *Nox4* mRNA expression was significantly increased after CoCl_2_ exposure as measured by real-time- PCR (Fig 1C). CoCl_2_ also increased the Nox4 protein level as measured by western blot (Fig 1D).

### Role of Nox4 in hypoxia-induced apoptosis in HK-2 cells

**Fig 2:**
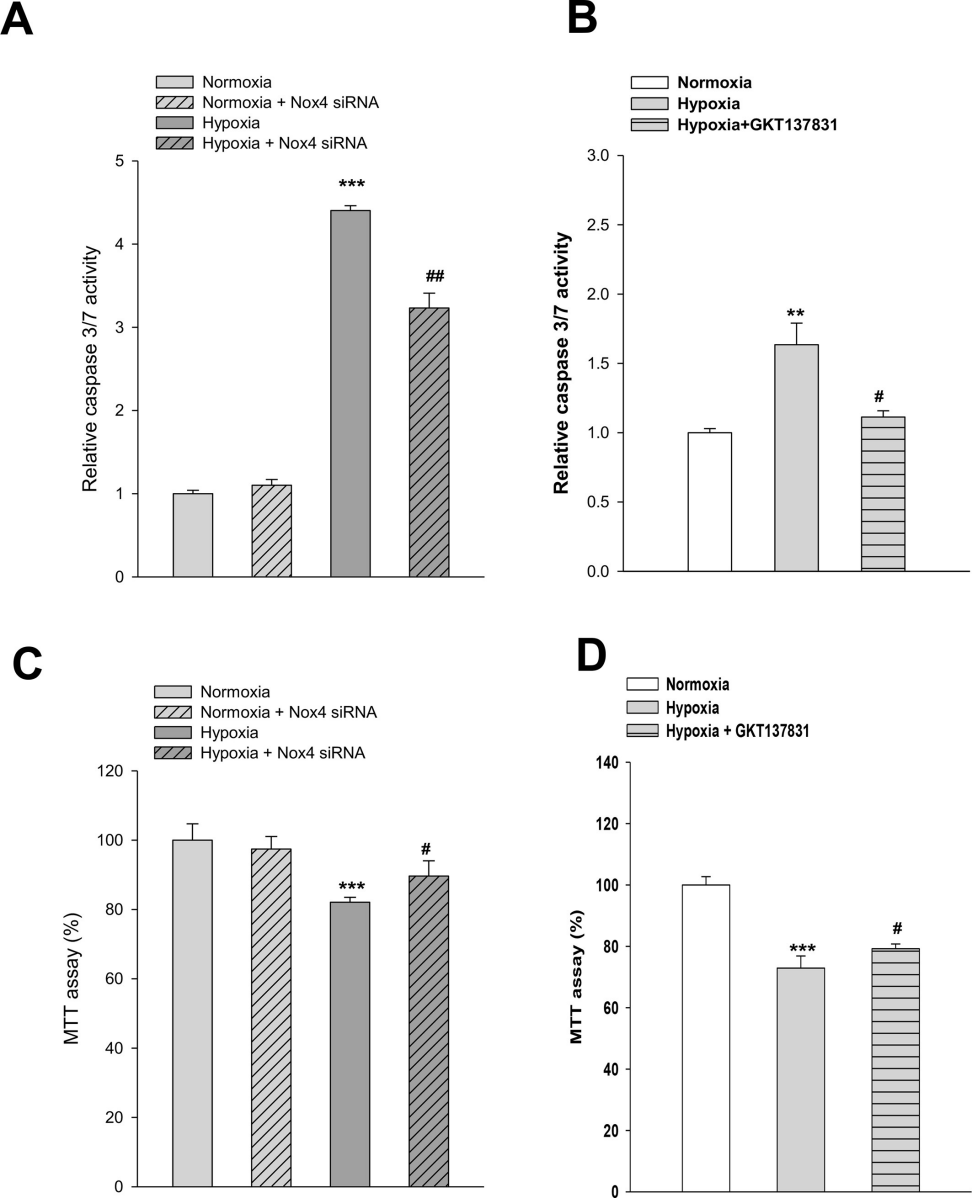
Nox4 is involved in hypoxia-induced apoptosis and cellular survival in HK-2 cells. HK-2 cells were cultured to 70–80% confluence, and CoCl_2_ was then added. Cells were incubated with CoCl_2_ at the indicated concentrations for 24 h. Apoptosis of HK-2 cells was assayed based on caspase 3/7 activity. **(A)** The effects of silencing of *Nox4* expression with siRNA; (**B**) pharmacologic inhibition of Nox1/4 with GKT137831. HK-2 cell viability was assayed using the MTT assay. (**C**) Silencing of *Nox4* expression with siRNA; (**D**) pharmacologic inhibition of Nox1/4 with GKT137831. Data represent means ± SD; n = 5; ^**^p < 0.01,^***^p < 0.001 vs. control and ^*#*^*p* < 0.05, ^#*#*^*p* < 0.01, ^###^*p*<0.001 vs. hypoxia alone.

To determine the role of Nox4 in hypoxia-induced apoptosis, cells were infected with *Nox4*-targeting or control small-interfering RNA (siRNA). The efficiency of Nox4 knockdown showed in S3 Fig. *Nox4* gene silencing blunted hypoxia-induced apoptosis (Fig 2A) as determined by caspase 3/7 activity. To reaffirm the results, we treated the cells with the specific Nox1/4 inhibitor GKT137831, finding that GKT137831 also attenuated caspase 3/7 activation (Fig 2B). In addition, Nox4 knockdown increased the survival of HK-2 cells in response to hypoxia as assessed using the MTT assay (Fig 2C). Pretreatment with GKT137831 also showed the renoprotective effects observed with *Nox4* silencing (Fig 2D).

### Effects of Nox4 inhibition on hypoxia-induced cellular ROS levels

**Fig 3:**
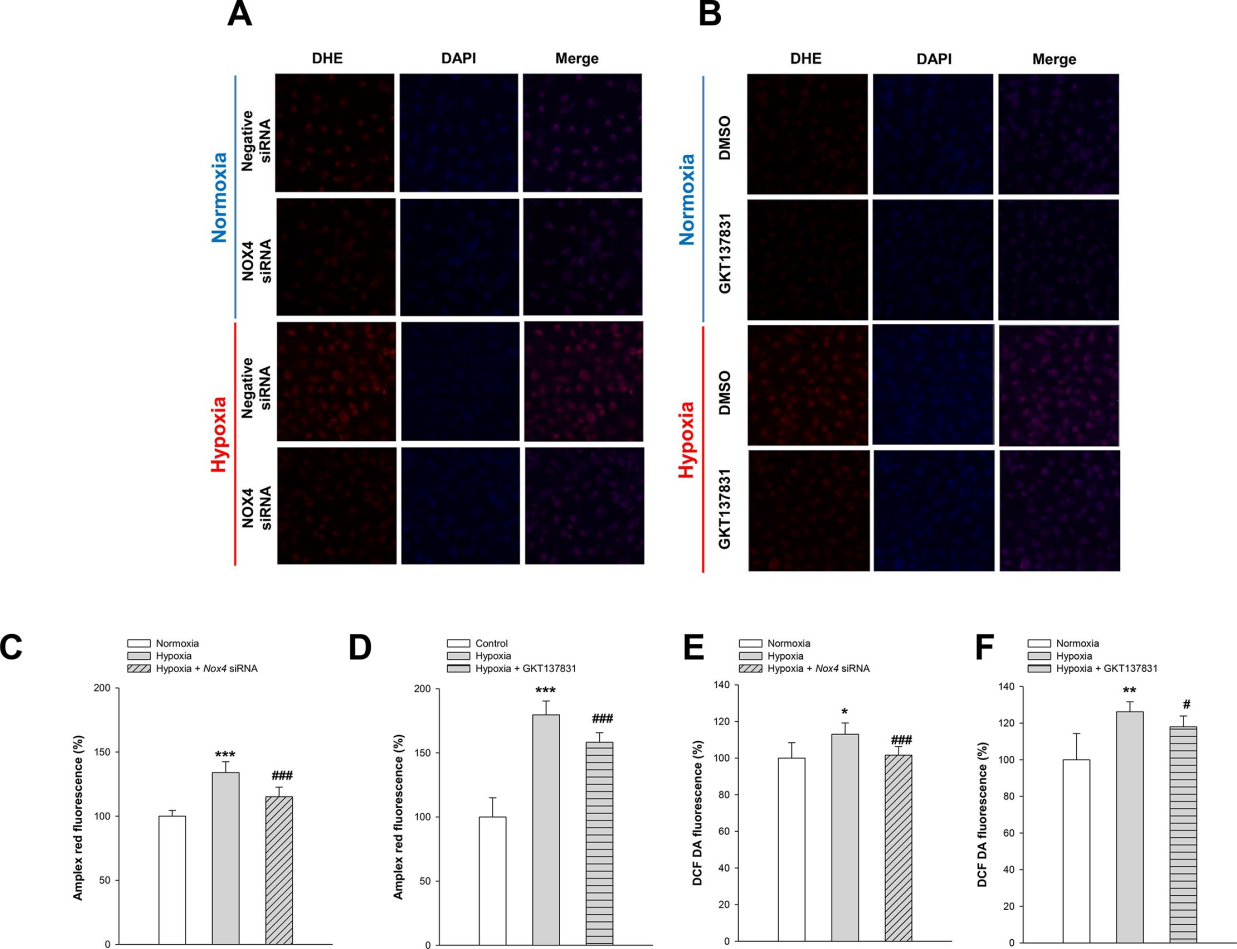
Effects of Nox4 inhibition on hypoxia-induced ROS generation. HK-2 cells were exposed to hypoxia for 24 hr. Confocal microscopy images of cells subjected to dihydroethidium (DHE) staining with and without *Nox4* knockdown (**A**) or treated with and without GKT137831 (**B**). Levels of H_2_O_2_, a product of Nox4, were measured by the Amplex red assay with and without *Nox4* knockdown (**C**) or treated with and without GKT137831 (**D**). Levels of ROS measured by DCF-DA with and without *Nox4* knockdown (**E**) or treated with and without GKT137831 (**F**). Data represent means ± SD; *p< 0.05,^**^p < 0.01,^***^p < 0.001 vs. control and ^*#*^*p* < 0.05, ^*##*^*p* < 0.01, ^###^*p*<0.001 vs. hypoxia alone.

We evaluated superoxide production after hypoxia exposure in HK-2 cells with and without *Nox4* knockdown or treated with and without GKT137831 using dihydroethidium (DHE) staining (Fig 3A and 3B). We also assessed hydrogen peroxide generation using an Amplex red assay and DCF-DA assay in cells with and without *Nox4* silencing or treated with and without GKT137831 after hypoxia exposure (Fig 3C–3F). Hypoxia induced a significant increase in ROS production 24 h after hypoxia exposure. Intriguingly, the extent of the effect of hypoxia on ROS levels was significantly suppressed by *Nox4* knockdown or GKT137831 pretreatment. The similar results were obtained when the hypoxia chamber was replace with CoCl_2_ (S4 Fig). This indicates that Nox4 is the source of the ROS mediating the hypoxia-induced HK-2 cell apoptosis.

### Effect of Nox4 inhibition on mitochondria in hypoxic HK-2 cells

**Fig 4:**
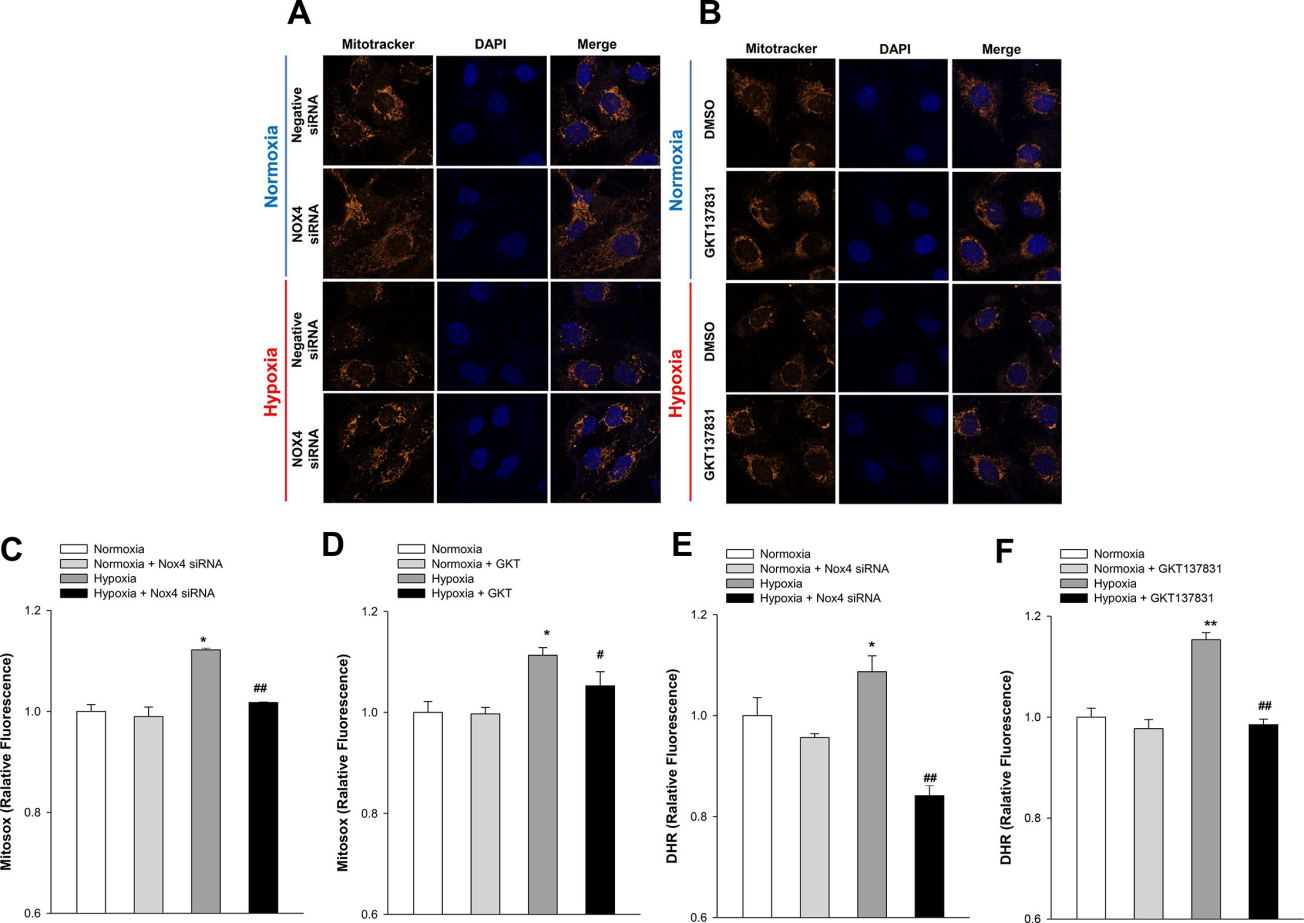
Effects of Nox4 inhibition on mitochondria in hypoxic HK-2 cells. HK-2 cells were exposed to hypoxia for 24 h. Confocal microscopy images of cells subjected to MitoTracker probes with and without *Nox4* knockdown (**A**) or treated with and without GKT137831 (**B**). Mitochondrial ROS formation was measured using MitoSOX with and without *Nox4* knockdown (**C**) or treated with and without GKT137831 (**D**). The intracellular ROS was measured using DHR with and without *Nox4* knockdown (**E**) or treated with and without GKT137831 (**F**). Data represent means ± SD; *p< 0.05,^**^p < 0.01,^***^p < 0.001 vs. control and ^*#*^*p* < 0.05, ^*##*^*p* < 0.01, ^###^*p*<0.001 vs. hypoxia alone.

We observed the effect of Nox4 inhibition on mitochondrial changes after hypoxia exposure using Mitotracker (Mitotracker Orange CMTMRos, Thermo Fisher Scientific, Inc., USA). Compared with the control group, the number of mitochondria was significantly reduced in the group exposed to hypoxia and recovered when Nox4 was blocked through Nox4 knockdown (Fig 4A) or GKT137831 pretreatment (Fig 4B). The mitochondrial ROS measured by MitoSOX (Invitrogen) significantly increased in hypoxia but decreased when exposed to hypoxia after Nox4 knockdown (Fig 3C) or GKT137831 pretreatment (Fig 3D). To compare mitochondrial and cytoplasmic ROS changes, cytoplasmic ROS were measured again using DHR 123. Similar to the results of mitochondrial ROS, it increased with exposure to hypoxia and decreased with Nox4 knockdown (Fig 3E) or GKT137831 pretreatment (Fig 3F). When the ratio of mitochondrial ROS and cytoplasmic ROS were checked, the ratio of mitochondria ROS was higher after Nox4 inhibition. As a result, we could guess that the degree of decreased of cytoplasmic ROS was higher than that of mitochondrial ROS through inhibition of Nox4.

### TGF-β1 induces Nox4 expression in hypoxic HK-2 cells

**Fig 5:**
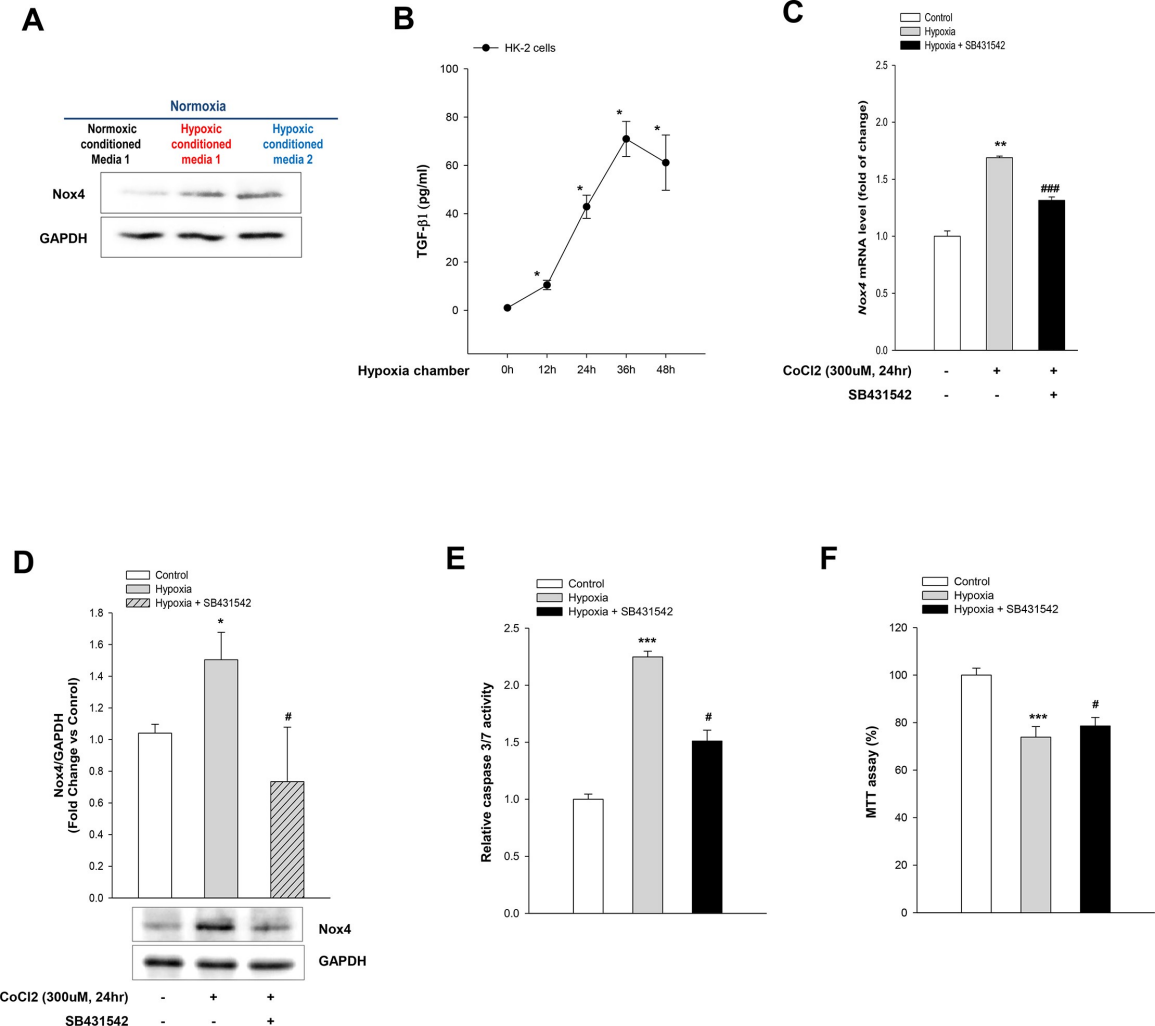
TGF-β1 mediates Nox4-dependent hypoxia-induced apoptosis of HK-2 cells. Western blot for Nox4 protein in hypoxia-conditioned media and in normoxic media (**A**). ELISA for TGF-β1 in HK-2 cells incubated under hypoxic conditions and normoxic conditions (**B**). Real-time PCR for *Nox4* mRNA with and without SB431542 (**C**). Western blot for Nox4 protein with or without SB41542 (**D**). Caspase 3/7 activity following treatment with and without SB431542 (**E**). MTT assay for cellular survival following treatment with and without SB431542 (**F**). Data represent means ± SD; *p< 0.01,^**^p < 0.05,^***^p < 0.001 vs. control and ^*#*^*p* < 0.05, ^*##*^*p* < 0.01, ^###^*p*<0.001 vs. hypoxia alone.

Culture media were collected from HK-2 cells that were exposed to hypoxia for 24 h and then added to normoxic HK-2 cells to examine whether hypoxia directly promote Nox4 expression in HK-2 cells or influences through a mediator as previously reported [23]. The Nox4 protein expression level was estimated 24 h later. Nox4 protein of normoxic HK-2 cells grown in hypoxia-conditioned culture media significantly increased compared with HK-2 cells grown in normoxic culture media in a volume-dependent manner (Fig 5A). This means that hypoxic HK-2 cells release soluble mediators that induce Nox4 expression [23]. Hypoxia is well known to cause the upregulation of TGF-β1 [23, 24]. The TGF-β1 levels in hypoxic HK-2 cells were measured by ELISA to know whether HK-2 cells exposed to hypoxia secrete TGF-β1 [23]. There was a significant increase in active TGF-β1 within 12 h under hypoxic conditions. TGF-β1 levels peaked 36 h after the hypoxia and then decreased; however, their levels remained high for 48 h compared with media from HK-2 cells cultured under normoxic conditions (Fig 5B).

To further confirm whether TGF-β1 induces Nox4 expression in hypoxia, HK-2 cells were grown with and without the TGF-β1 type 1 tyrosine kinase inhibitor, SB431542. Treatment with SB431542 significantly decreased Nox4 expression with real-time PCR and western blotting (Fig 5C and 5D).

To examination the effect of TGF-β1 on cellular apoptosis and survival in hypoxia, HK-2 cells were pretreated with and without SB431542 and then incubated under normoxia and hypoxia. Caspase 3/7 activity was measured and an MTT assay was performed. The caspase 3/7 activity was significantly attenuated and cellular survival significantly increased by pretreatment with SB431542 under hypoxia (Fig 5E and 5F).

### The Smad pathway mediates TGF-β1-induced Nox4 expression in hypoxic cell injury

**Fig 6:**
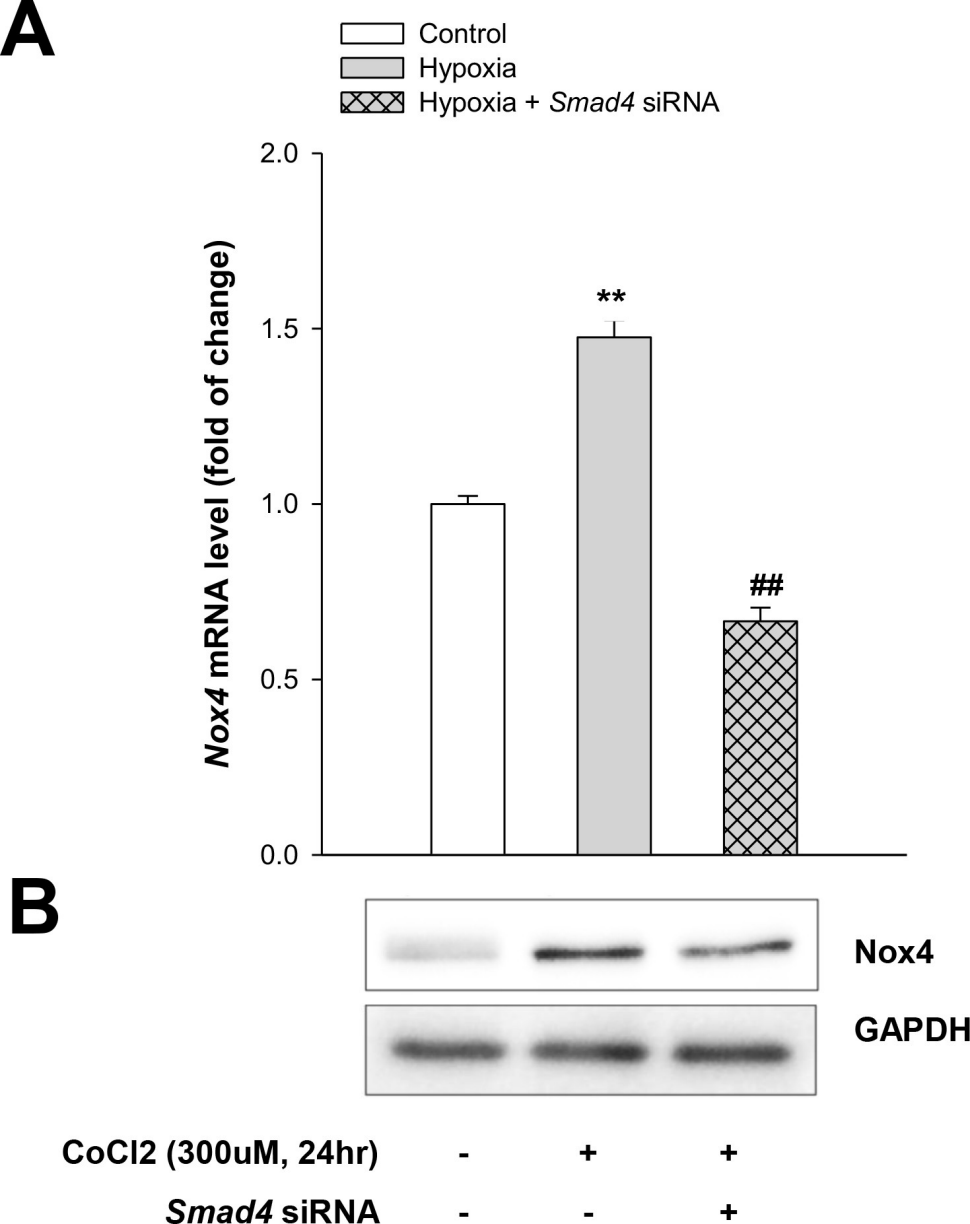
Smad pathway mediates TGF-β1 induction of Nox4 expression in hypoxic cell injury. Quantitative real-time PCR for Nox4 mRNA with and without knockdown of *Smad4* (**A**). Western blot for Nox4 protein with and without knockdown of *Smad4* (**B**). Data represent means ± SD; ^**^p < 0.05 vs. control and ^*##*^*p* < 0.01 vs. hypoxia alone.

To examine whether Smad-dependent signaling pathway is involved in Nox4 expression in TGF-β1-induced hypoxic injury, HK-2 cells were infected with *Smad4* siRNA or control siRNA. The gene silencing efficacy of the *Smad4* siRNA was confirmed by western blot, with Smad4 gene silencing significantly decreasing TGF-β1-induced Nox4 expression measured by mRNA transcripts and western blot (Fig 6A and 6B).

### Mitogen-activated protein kinases (MAPKs) pathways mediate redox-sensitive, hypoxia-induced apoptosis in HK-2 cells

**Fig 7:**
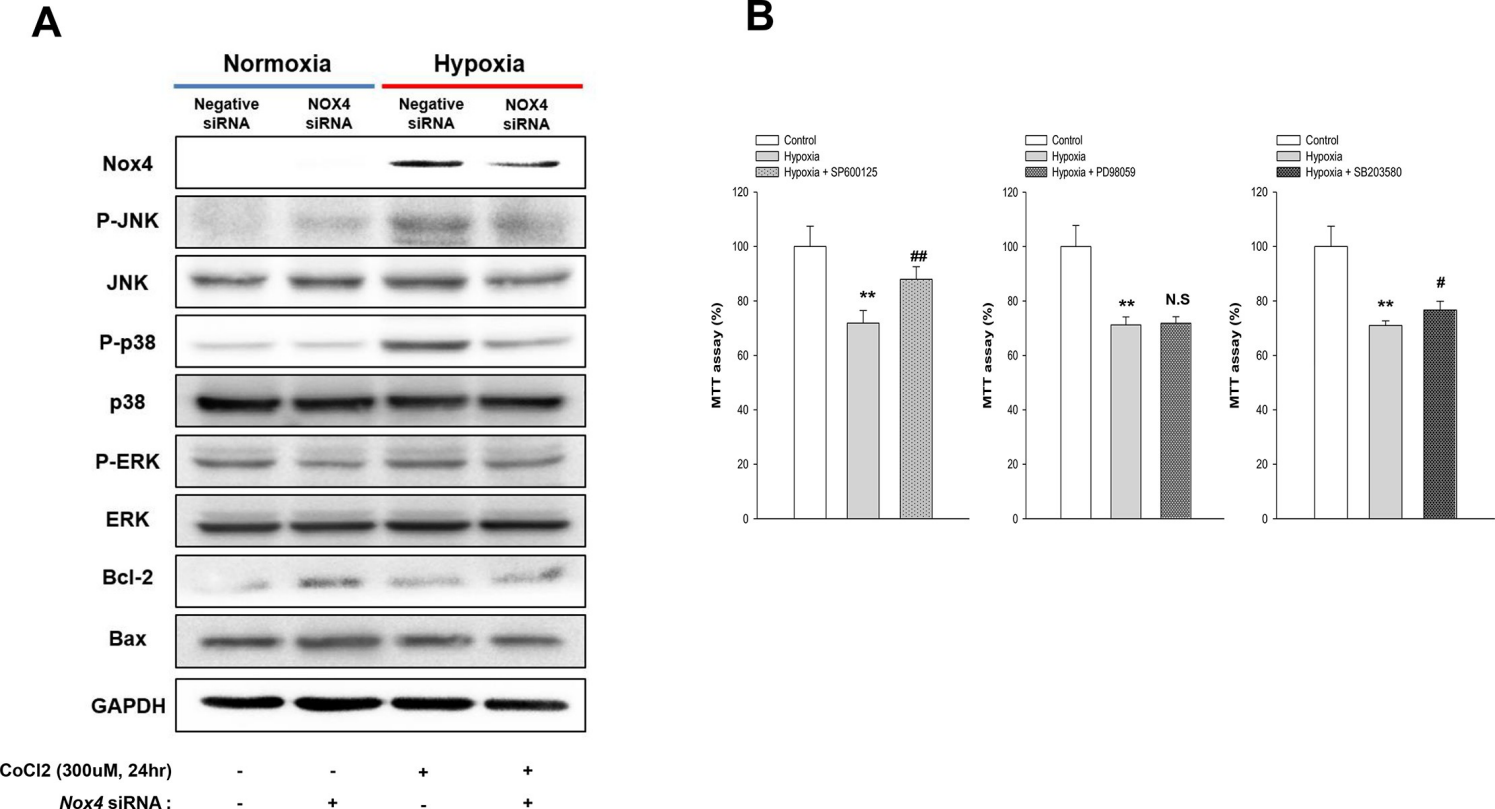
Mitogen-activated protein kinases (MAPKs) pathways involved in Nox4 -induced HK-2 cell apoptosis in hypoxia. Immunoblotting of Nox4-dependent intracellular signaling in CoCl_2_ treated HK-2 cells with and without *Nox4* knockdown (**A**). HK-2 cell viability was assayed using the MTT assay with and without pharmacologic inhibition of JNK, p38 and ERK with SP600125, SB203580 and PD98059, respectively under hypoxic condition (**B**). ^**^p < 0.05 vs. control and ^*#*^*p* < 0.05, ^*##*^*p* < 0.01 vs. hypoxia alone.

We analyzed activation of the redox sensitive MAPK pathways (p38, JNK, and ERK) to identify the signaling mechanisms underlying hypoxia and Nox4 medicated apoptosis. Hypoxia (300uM of CoCl_2_) led to increased phosphorylation of p38, JNK and ERK after 24 h. To determine the role of Nox4 in MAPK activation, cells were infected with *Nox4*-targeting or control siRNA. *Nox4* gene silencing significantly reduced hypoxia-induced phosphorylation of JNK and p38. The levels of Bax decreased and Bcl-2 increased (Fig 7A). We further confirmed the relation between hypoxia and JNK, p38 and ERK pathways by inhibition of them with SP600125, SB203580 and PD98059, respectively. Notably, inhibition of JNK and p38 improved cellular survival (Fig 7B).

### Pretreatment of GKT137831 attenuated hypoxia induced acute kidney injury and oxidative stress in rats

To assay changes in renal function after ischemic injury, we measured blood urea nitrogen (BUN) and creatinine in serum of ischemic / reperfusion (I/R) injured rats. Serum BUN and creatinine levels were not changed in the Group 2 (GKT treatment + sham operation) compared to Group 1. In Group 3 (I/R operation) serum BUN and creatinine levels were significantly increased when compared to Group 1 (control, sham operation). However, in Group 4 (GKT pretreatment + I/R operation), the damage of renal function due to ischemia was improved (Table 1).

**Table 1:** Renal function in the study groups (mean ± SD).

		Groups		
Parameter	G1 (n = 5) Sham operation	G2 (n = 5) Sham operation + GKT137831 (10 mg/kg)	G3 (n = 5) I/R operation	G4 (n = 5) I/R operation + GKT137831 (10 mg/kg)
**BUN (mg/dL)**	22.9 ± 2.7	18.6 ± 4	79.7 ± 20**	27.3 ± 8.1^###^
**Cr (mg/dL)**	0.2 ± 0.00	0.2± 0.1	0.85 ± 0.5	0.21 ± 0.08

Abbreviations: G1, group 1; G2, group2; G3, group 3; G4, group 4; BUN, blood urea nitrogen; Cr, creatinine. The data are the mean ± SD (n = 5).

**p < 0.01 versus the control and

^###^*p* < 0.001 versus the I/R operation group.

**Fig 8:**
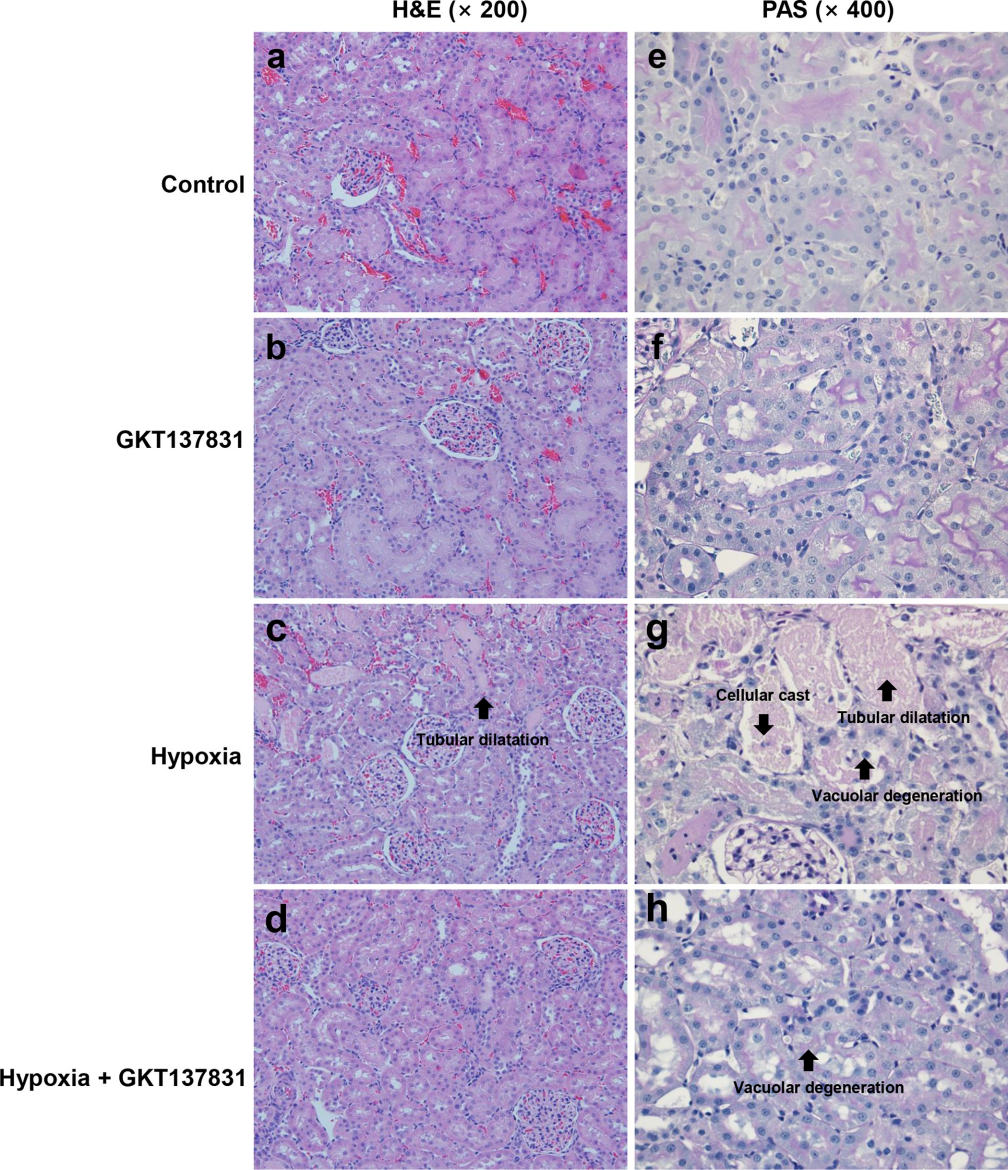
Renal histopathology of ischemia / reperfusion injured rats with or without GKT137831 pretreatment. Representative photomicrographs of hematoxylin and eosin (H&E) stained kidney sections (**A-D**) and Periodic acid-Schiff (PAS) stained kidney sections (**E-F**) of tissues isolated 24 h after I/R operation with or without GKT137831 pretreatment. Magnifications x 200 and x 400.

Histologic changes were analyzed in control and experimental rats. There was no significant difference between Group1 and Group 2, but tubular dilatation, cellular casts, loss of tubular brush borders, vacuolar degeneration and tubular epithelial cell shedding were observed in Group 3. In Group 4, tubular damage was restored when compared to Group 3 (Fig 8A–8F).

**Fig 9:**
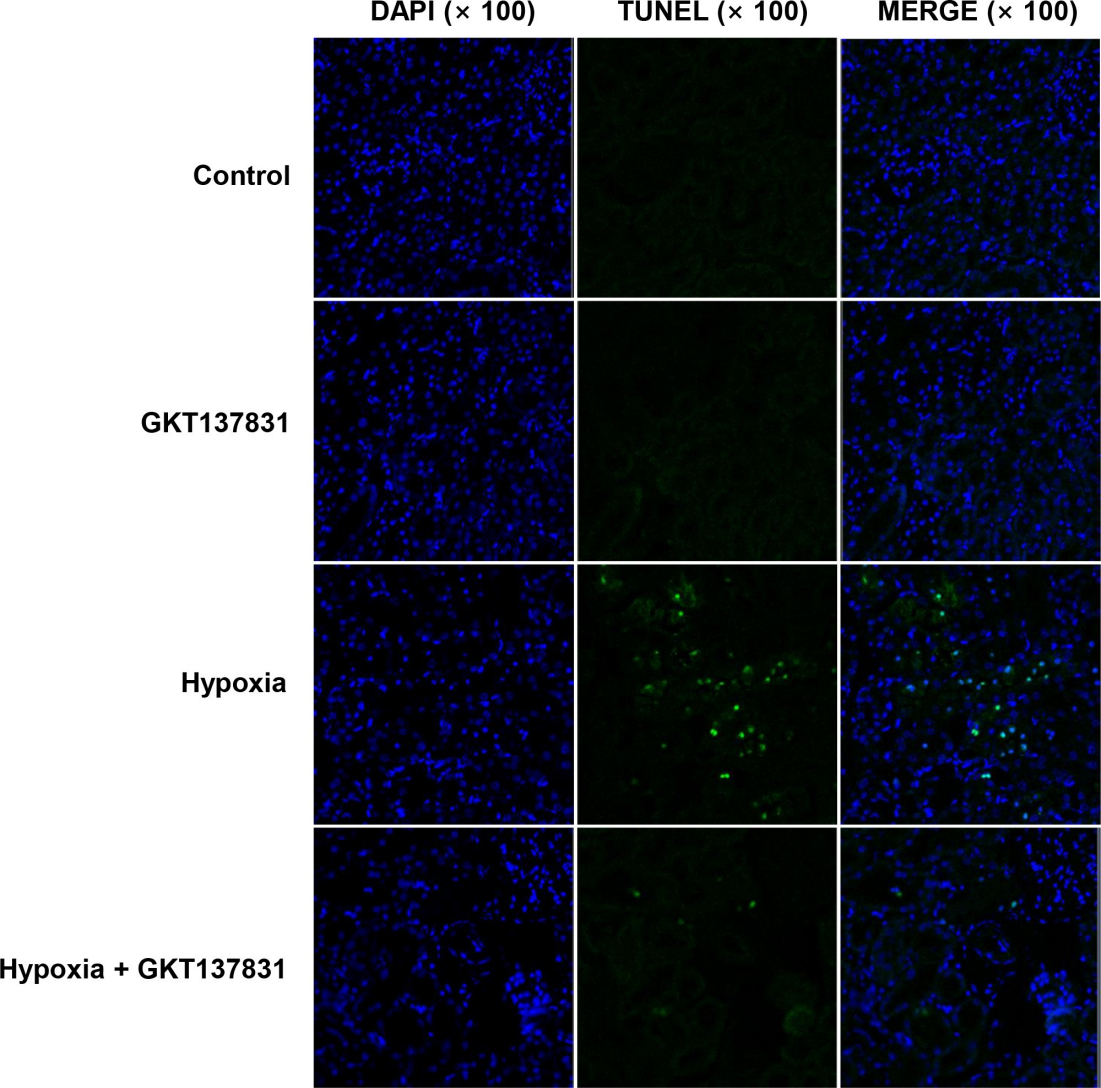
Apoptotic kidney cells in ischemia / reperfusion injured rats reduced after GKT137831 pretreatment. TUNEL staining in sham operated rats or I/R operated rats with or without GKT137831 pretreatment. Representative confocal micrographs of TUNEL stained kidney sections at 24 h after I/R injury.

To detect apoptotic changes in in kidney cells, we performed TUNEL assay in control and experimental rats. In Group 3, there was a significant number of positive cells in the TUNEL staining, whereas in Group 4, the number of TUNEL positive cells was significantly decreased (Fig 9)

## Discussion

Although it is located ubiquitously in cells, Nox4 is considered a renox agent because it is predominantly present in the kidney. The primary role of Nox4 lies in the generation of free radicals, especially hydrogen peroxide. Given the importance of ROS in hypoxia induced AKI, we hypothesized that Nox4 inhibition could prevent hypoxia induced AKI.

HK-2 cell apoptosis driven by hypoxia-induced oxidative stress involves the TGF-β/Smad/Nox4 signaling pathway in this study. Our results show that HK-2 cell apoptosis, characterized by caspase 3/7 activity, is regulated through Nox4-generated ROS. By using a pharmacological inhibitor of TGF-β1 signaling, we demonstrated that TGF-β1-induced HK-2 cell apoptosis acts through the TGF-β receptor 1/Smad/Nox4 signaling cascade. TGF-β1 enhanced the expression of Nox4 and the TGF-β1 receptor blocker SB431542 decreased Nox4 expression.

Although Nox4 generated ROS, its function in the cell is not entirely clear and it may be regulated by multiple factors according to the cell type and variant of Nox homolog(s) present in the cell [25]. Previous studies have reported that in pathologic conditions, overexpression of Nox4 in podocytes and mesangial cells leads to renal fibrosis and chronic kidney disease through increasing cellular ROS level. [13,26,27]. Even though renal tubular epithelial cell injury is a key feature of the initiation of AKI and overexpression of Nox4 in kidney epithelial cells has been noted, its role in AKI has not been well characterized in these cells. A few studies including previous author’s studies reported Nox4 involvement in apoptosis in human proximal tubule cells in several AKI models [14–16, 28,29]. Song et al. reported before that diphenylene iodonium (DPI) abolish hypoxia induced apoptosis of HK-2 cells [14]. But DPI is known to nonspecifically reduce Nox activity by inhibition of flavoprotein. So this is the first study in our knowledge to describe the role of Nox4 in HK-2 cells in hypoxic condition. There is a study that reported the contradictory results of the author’s findings on the role of Nox4 in hypoxia. Nlandu-Khodo et al [30], reported that Nox4 knockout mice showed increased tubular cell death during acute ischemia/reperfusion injury with decreased Nrf2 protein expression. We performed *in-vivo* study with wild type rats and GKT/137831 for Nox4 inhibition instead of Nox4 knockout mouse. In our study, in contrast to Nlandu-Khodo et al, kidney damage due to I/R was alleviated by GKT137831 pretreatment. ROS are supposed to have dual roles in biological systems. Even though maintaining a basal level of ROS is crucial for life, immoderate concentrations of ROS can destroy balance and cause adverse effects. Thus, whether Nox4 plays a good or bad role in body might be determined by how much of the appropriate ROS is present in various cells and different condition [31]. Different levels of oxidative stress could lead to different effects on kidney injury in hypoxia. AKI due to hypoxia not present only in the ischemia/reperfusion injury but in other ischemic or non-ischemic injury can occur, further in-vivo study in various settings is needed.

In our study, not only cytoplasmic but also mitochondrial ROS were reduced by Nox4 inhibition. It is known that Nox4 directly or indirectly affects mitochondrial ROS. With direct influence, Nox4 is located not only cytoplasmic membrane but also mitochondrial membrane. Mitochondrial Nox4 is known as one of the important source of mitochondrial ROS in diabetes [32] and aging associated cardiovascular disease [33]. On the other hand, indirectly H_2_O_2_ produced by cytoplasmic Nox4 activates Nox2, which increases the ROS of mitochondria and activates the VEGF signaling in endothelial cells [34]. Crosstalk between mitochondria and NADPH oxidases has been studied as an interesting subject [35]. Even though further studies are needed to clarify the relationship, the results of this study may be a good basis for explaining the relationship between Nox4 and mitochondria in hypoxia.

Among several reported pathways that related in TGF-β1 induced the expression of Nox4 (Smad pathway, PI3K pathway, MAPK pathways and RhoA/ROCK pathway) [36], TGF-β1/Smad signaling pathway was involved in this study. The Smads accumulate in the nucleus as the Smad2-3/Smad4 complex induce Nox4 expression by binding in the Nox4 gene promoter region [37]. It is well known that TGF-β induced Nox4 associated fibrotic response in many cells [36] but a few studies including author’s previous studies reported the role of TGF-β/Nox4 signaling in cellular apoptosis [15, 38].

In this study, the JNK and p38 pathways were critical in mediating hypoxia-induced HK-2 cell apoptosis. Although phosphorylation of JNK, ERK and p38 increased after hypoxia exposure, only phosphorylation of JNK and p38 decreased after knockdown of Nox4. Our results are consistent with previous studies that suggest that p38 and JNK are involved in apoptosis and ERK is involved in cellular survival in ischemia/reperfusion kidney injury [39]. Because MAPK pathway has various effects on different cells and even in the same cells shows different changes depending on the situation, it is necessary to study the change pattern of MAPKs according to the change of amount of ROS.

**Fig 10:**
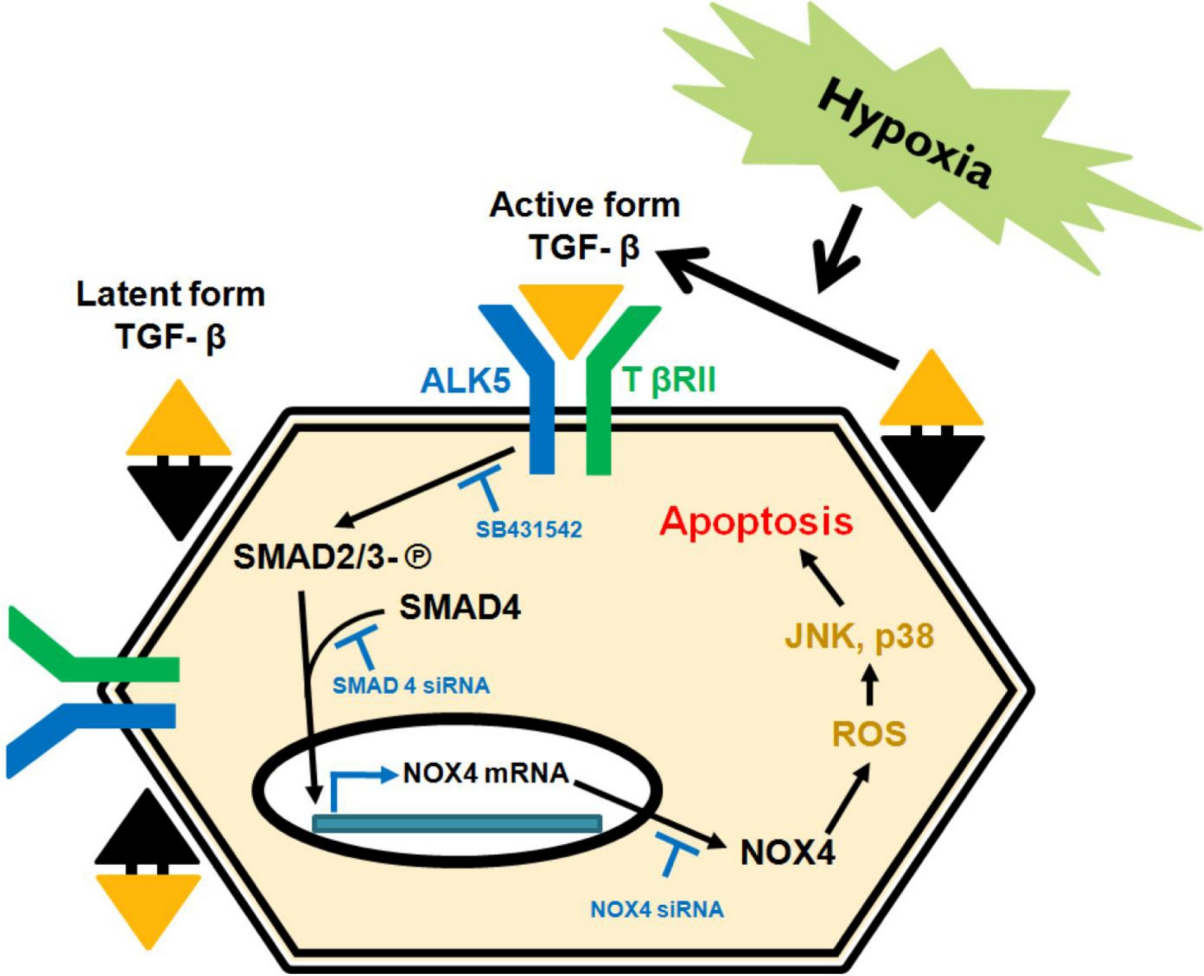
Schematic diagram of hypoxia-induced acute kidney injury enhanced by oxidative stress driven by activation of Nox4 expression in HK-2 cells. Hypoxia can induce the apoptosis of proximal tubular cells by increasing oxidative stress driven by TGF-β/Smad signaling-mediated Nox4 expression. Inhibition of Nox4 by either genetic knockdown of *NOX4* or treatment with GKT137831 (a specific Nox1/4 inhibitor) attenuated hypoxia-induced proximal tubular cell apoptosis by reducing the phosphorylation of MAPKs (notably the p38 and JNK MAPK subfamily).

Our findings show that Nox4 participates in the pathogenesis of HK-2 cell apoptosis in hypoxia. Our present study suggest that AKI driven by hypoxia-induced oxidative stress involves the TGF-β/Smad/Nox4/MAPK signaling pathway to drive redox signaling in HK-2 cells (Fig 10). Our data also strongly suggest that the renoprotective effects of Nox4 inhibition involve Nox4-dependent reductions in oxidative damage and apoptosis. Thus, we propose that hypoxia upregulates the expression of Nox4 via the TGF-β/Smad pathway and increases ROS production and therapies targeting Nox4 may be effective against hypoxia-induced acute kidney injury.

## Supporting information

S1 Fig Effects of hypoxia on cell viability. The viability of HK-2 cells treated with CoCl_2_ for 24 h was measured by MTT (**A**). Western blot for HIF-1α protein with and without CoCl_2_ (**B**). Data represent means ± SD; *p< 0.05,^**^p < 0.01,^***^p < 0.001 at each time point vs. control.
(TIF)

S2 Fig Nox4 knockdown efficiency analysis. The efficiency of Nox4 knockdown was confirmed by measuring Nox4 mRNA expression and the amount of Nox4 protein by qRT-PCR and western blotting, respectively.
(TIF)

S3 Fig S1 Fig. **Scheme for the pretreatment of GKT137831 on ischemic reperfusion injury model** GKT137831 (10mg/kg) or normal saline (5ml/kg) was administered orally once a day during four days prior to the ischemic injury (45min) and once after ischemic injury. The rats were scarified 24 hours after ischemic injury.
(TIF)

S4 Fig Effects of Nox4 inhibition on hypoxia-induced ROS generation. HK-2 cells were exposed to CoCl_2_. Confocal microscopy images of cells subjected to dihydroethidium (DHE) staining with and without *Nox4* knockdown (**A**) or treated with and without GKT137831 (**B**). Levels of H_2_O_2_, a product of Nox4, were measured by the Amplex red assay with and without *Nox4* knockdown (**C**) or treated with and without GKT137831 (**D**). Levels of ROS measured by DCF-DA with and without *Nox4* knockdown (**E**) or treated with and without GKT137831 (**F**). Data represent means ± SD; *p< 0.01,**p < 0.05,***p < 0.001 vs. control and ^*#*^*p* < 0.05, ^*##*^*p* < 0.01, ^###^*p*<0.001 vs. hypoxia alone.
(TIF)

## References

[1] Mehta RL, Kellum JA, Shah SV, Molitoris BA, Ronco C, Warnock DG, et al Acute Kidney Injury Network: report of an initiative to improve outcomes in acute kidney injury. Crit Care 2007;11(2):R31 10.1186/cc5713 17331245 PMC2206446

[2] Sawhney S, Fraser SD. Epidemiology of AKI: Utilizing Large Databases to Determine the Burden of AKI. Adv Chronic Kidney Dis. 2017;24(4):194–204. 10.1053/j.ackd.2017.05.001 28778358 PMC5648688

[3] Nath KA, Norby SM. Reactive oxygen species and acute renal failure. Am J Med. 2000; 109(8):665–78. 10.1016/s0002-9343(00)00612-4 11099687

[4] Bonventre JV, Yang L. Cellular pathophysiology of ischemic acute kidney injury J Clin Invest. 2011;121(11):4210–21. 10.1172/JCI45161 22045571 PMC3204829

[5] Malek M, Nematbakhsh M. Renal ischemia/reperfusion injury; from pathophysiology to treatment. J Renal Inj Prev 2015;4(2):20–7. 10.12861/jrip.2015.06 26060833 PMC4459724

[6] Kalogeris T, Baines CP, Krenz M, Korthuis RJ. Cell biology of ischemia/reperfusion injury. Int Rev Cell Mol Biol 2012;298:229–317. 10.1016/B978-0-12-394309-5.00006-7 22878108 PMC3904795

[7] Riedemann NC, Ward PA. Complement in ischemia reperfusion injury. Am J Pathol 2003;162(2):363–7. 10.1016/S0002-9440(10)63830-8 12547694 PMC1851148

[8] Granger DN, Kvietys PR. Reperfusion injury and reactive oxygen species: The evolution of a concept. Redox Biol. 2015;6:524–551. 10.1016/j.redox.2015.08.020 26484802 PMC4625011

[9] Karim AS, Reese SR, Wilson NA, Jacobson LM, Zhong W, Djamali A. Nox2 is a mediator of ischemia reperfusion injury. Am J Transplant. 2015;15(11):2888–99. 10.1111/ajt.13368 26104383 PMC4636908

[10] McCord JM. Oxygen-derived free radicals in postischemic tissue injury. N Engl J Med 1985;312(3):159–63. 10.1056/NEJM198501173120305 2981404

[11] Paller MS, Hoidal JR, Ferris TF. Oxygen free radicals in ischemic acute renal failure in the rat. J Clin Invest 1984;74(4):1156–64. 10.1172/JCI111524 6434591 PMC425281

[12] Nlandu Khodo S, Dizin E, Sossauer G, Szanto I, Martin PY, Feraille E, et al NADPH-oxidase 4 protects against kidney fibrosis during chronic renal injury. J Am Soc Nephrol 2012;23(12):1967–76. 10.1681/ASN.2012040373 23100220 PMC3507365

[13] Jha JC, Gray SP, Barit D, Okabe J, El-Osta A, Namikoshi T et al Genetic targeting or pharmacologic inhibition of NADPH oxidase nox4 provides renoprotection in long-term diabetic nephropathy. J Am Soc Nephrol 2014;25(6):1237–54. 10.1681/ASN.2013070810 24511132 PMC4033375

[14] Song H, Han IY, Kim Y, Kim YH, Choi IW, Seo SK et al The NADPH oxidase inhibitor DPI can abolish hypoxia-induced apoptosis of human kidney proximal tubular epithelial cells through Bcl2 up-regulation via ERK activation without ROS reduction. Life Sci. 2015;126:69–75. 10.1016/j.lfs.2015.02.004 25744050

[15] Jeong BY, Park SR, Cho S, Yu SL, Lee HY, Park CG et al TGF-β-mediated NADPH oxidase 4-dependent oxidative stress promotes colistin-induced acute kidney injury. J Antimicrob Chemother 2018; 73(4):962–72. 10.1093/jac/dkx479 29329393

[16] Jeong BY, Lee HY, Park CG, Kang J, Yu SL, Choi DR et al Oxidative stress caused by activation of NADPH oxidase 4 promotes contrast-induced acute kidney injury. PLoS One 2018;13(1):e0191034 10.1371/journal.pone.0191034 29329317 PMC5766150

[17] Gorin Y, Wauquier F. Upstream regulators and downstream effectors of NADPH oxidases as novel therapeutic targets for diabetic kidney disease. Mol cells 2015;38(4):285–96. 10.14348/molcells.2015.0010 25824546 PMC4400302

[18] Piret JP, Mottet D, Raes M, Michiels C. CoCl_2_, a chemical inducer of hypoxia-inducible factor-1, and hypoxia reduce apoptotic cell death in hepatoma cell line HepG2. Ann N Y Acad Sci 2002;973:443–7. 10.1111/j.1749-6632.2002.tb04680.x 12485908

[19] Pourgholami MH, Cai ZY, Badar S, Wangoo K, Poruchynsky MS, Morris DL. Potent inhibition of tumoral hypoxia-inducible factor 1alpha by albendazole BMC Cancer 2010;10:143 10.1186/1471-2407-10-143 20398289 PMC2873385

[20] Yuan Y, Hilliard G, Ferguson T, Millhorn DE.Cobalt inhibits the interaction between hypoxia-inducible factor-alpha and von Hippel–Lindau protein by direct binding to hypoxia-inducible factor-alpha J Biol Chem. 2003;278(18):15911–6. 10.1074/jbc.M300463200 12606543

[21] Gorin Y. Nox4 as a potential therapeutic target for treatment of uremic toxicity associated to chronic kidney disease. Kidney international 2013;83(4):541–3. 10.1038/ki.2012.434 23538692 PMC3616333

[22] Malek M, Nematbakhsh M. The preventive effects of diminazene aceturate in renal ischemia/reperfusion injury in male and female rats. Adv Prev Med. 2014; 2014():740647 10.1155/2014/740647 25478235 PMC4247946

[23] Moon YM, Kang HJ, Cho JS, Park IH, Lee HM. Nox4 mediates hypoxia-stimulated myofibroblast differentiation in nasal polyp-derived fibroblasts. Int Arch Allergy Immunol. 2012;159(4):399–409. 10.1159/000337658 22846744

[24] Zhou G, Dada LA, Wu M, Kelly A, Trejo H, Zhou Q, et al Hypoxia-induced alveolar epithelial-mesenchymal transition requires mitochondrial ROS and hypoxia-inducible factor 1. Am J Physiol Lung Cell Mol Physiol. 2009;297(6):L1120–1130. 10.1152/ajplung.00007.2009 19801454 PMC2793183

[25] Bondi CD, Manickam N, Lee DY, Block K, Gorin Y, Abboud HE, et al https://www.ncbi.nlm.nih.gov/pubmed/?term=Barnes%20JL%5BAuthor%5D&cauthor=true&cauthor_uid=19926889NAD(P)H oxidase mediates TGF-beta1-induced activation of kidney myofibroblasts. Am Soc Nephrol 2010;21(1):93–102. 10.1681/ASN.2009020146 19926889 PMC2799274

[26] Gorin Y, Wauquier F. Upstream regulators and downstream effectors of NADPH oxidases as novel therapeutic targets for diabetic kidney disease. Mol Cells 2015;38(4): 285–96. 10.14348/molcells.2015.0010 25824546 PMC4400302

[27] Gorin Y. Nox4 as a potential therapeutic target for treatment of uremic toxicity associated to chronic kidney disease. Kidney Int 2013;83(4):541–3. 10.1038/ki.2012.434 23538692 PMC3616333

[28] Yao M, Gao F, Wang X, Shi Y, Liu S, Duan H. Nox4 is involved in high glucose-induced apoptosis in renal tubular epithelial cells via Notch pathway. Mol Med Rep 2017;15(6):4319–25. 10.3892/mmr.2017.6516 28487945

[29] Li Z, Sheng Y, Liu C, Li K, Huang X, Huang J, et al Nox4 has a crucial role in uric acid-induced oxidative stress and apoptosis in renal tubular cells. Mol Med Rep 2016;13(5):4343–8. 10.3892/mmr.2016.5083 27052425

[30] Nlandu-Khodo S, Dissard R, Hasler U, Schäfer M, Pircher H, Jansen-Durr P, et al NADPH oxidase 4 deficiency increases tubular cell death during acute ischemic reperfusion injury. Sci Rep 2016;6:38598 10.1038/srep38598 27924932 PMC5141508

[31] Brandes RP, Takac I, Schröder K. No superoxide—no stress?: Nox4, the good NADPH oxidase! Arterioscler Thromb Vasc Biol 2011;31(6):1255–7. 10.1161/ATVBAHA.111.226894 21593458

[32] Block K, Gorin Y, Abboud HE. subcellular localization of Nox4 and regulation in diabetes. Proc Natl Acad Sci U S A. 2009;25;106:14385–90. 10.1073/pnas.0906805106 19706525 PMC2732863

[33] Vendrov AE, Vendrov KC, Smith A, Yuan J, Sumida A, Robidoux et al NOX4 NADPH Oxidase-Dependent Mitochondrial Oxidative Stress in Aging-Associated Cardiovascular Disease.Antioxid Redox Signal. 2015;20;23:1389–409. 10.1089/ars.2014.6221. PMC4692134 26054376

[34] Kim YM, Kim SJ, Tatsunami R, Yamamura H, Fukai T, Ushio-Fukai M. ROS-induced ROS release orchestrated by Nox4, Nox2, and mitochondria in VEGF signaling and angiogenesis. Am J Physiol Cell Physiol. 2017;1;312:C749–C764. 10.1152/ajpcell.00346.2016 28424170 PMC5494593

[35] Dikalov S. Crosstalk between mitochondria and NADPH oxidases. Free Radic Biol Med. Free Radic Biol Med. 2011;1; 51:1289–1301 10.1016/j.freeradbiomed.2011.06.033 21777669 PMC3163726

[36] Liu RM, Desai LP. Reciprocal regulation of TGF-β and reactive oxygen species: A perverse cycle for fibrosis. Redox Biol. 2015;6:565–77 10.1016/j.redox.2015.09.009 26496488 PMC4625010

[37] Jiang F, Liu GS, Dusting GJ, Chan EC. NADPH oxidase-dependent redox signaling in TGF-β-mediated fibrotic responses. Redox Biol. 2014;2:267–72. 10.1016/j.redox.2014.01.012 24494202 PMC3909817

[38] Yan F, Wang Y, Wu X, Peshavariya HM, Dusting GJ, Zhang M et al Nox4 and redox signaling mediate TGF-β-induced endothelial cell apoptosis and phenotypic switch. Cell Death Dis. 2014 23;5:e1010 10.1038/cddis.2013.551 24457954 PMC4040700

[39] Luo F, Shi J, Shi Q, Xu X, Xia Y, He X. Mitogen-Activated Protein Kinases and Hypoxic/Ischemic Nephropathy. Cell Physiol Biochem. 2016;39(3):1051–67. 10.1159/000447812 27544204

