# Supplementary figures and images for: NADPH oxidase 4 mediates TGF-β1/Smad signaling pathway induced acute kidney injury in hypoxia
Source: PLoS One. 2019 Jul 18;14(7):e0219483. doi: 10.1371/journal.pone.0219483 (PMC6638919; doi:10.1371/journal.pone.0219483)

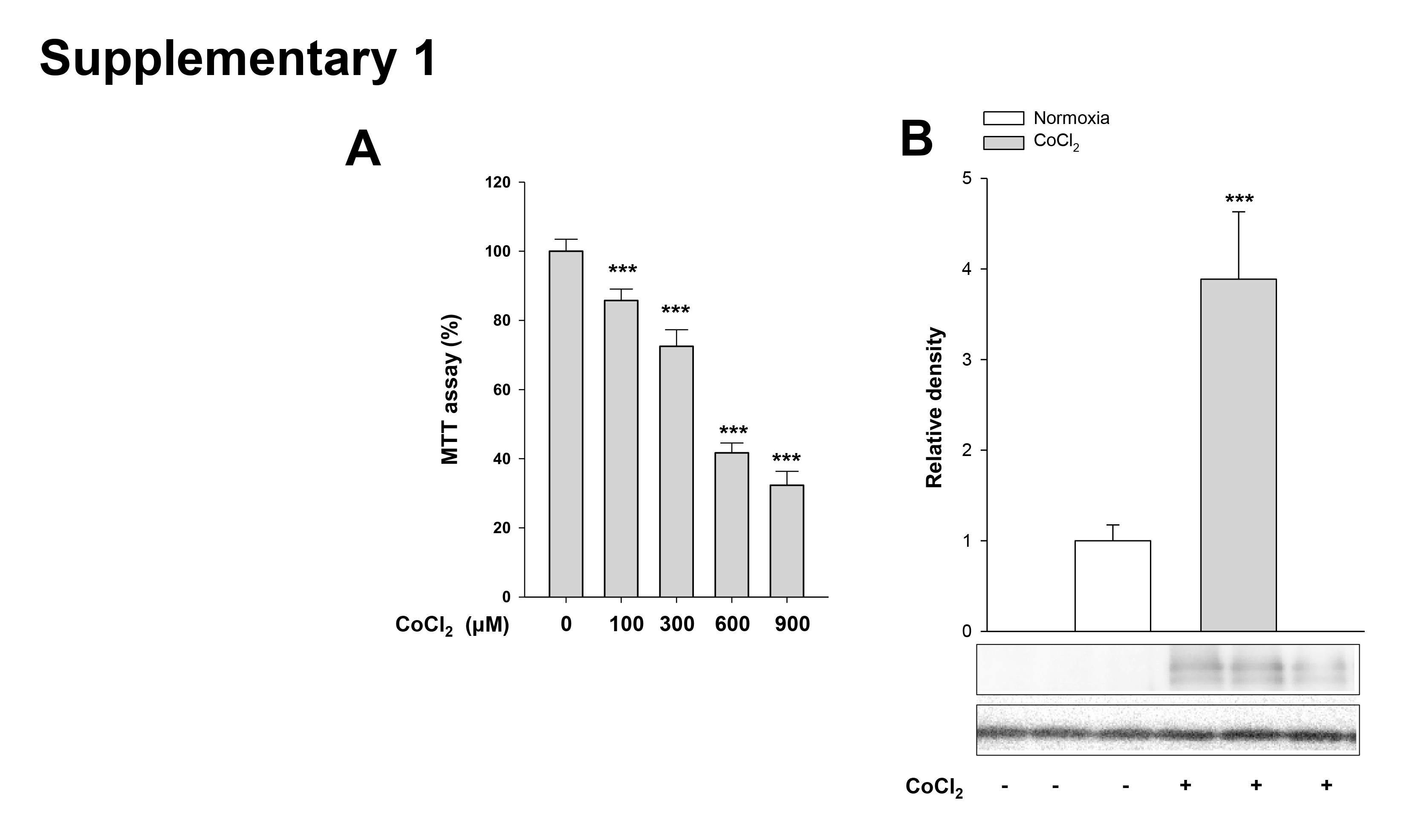

Supplement: S1 Fig — The viability of HK-2 cells treated with CoCl2 for 24 h was measured by MTT (A). Western blot for HIF-1α protein with and without CoCl2 (B). Data represent means ± SD; *p< 0.05,**p < 0.01,***p < 0.001 at each time point vs. control. (TIF) [file pone.0219483.s001.tif]

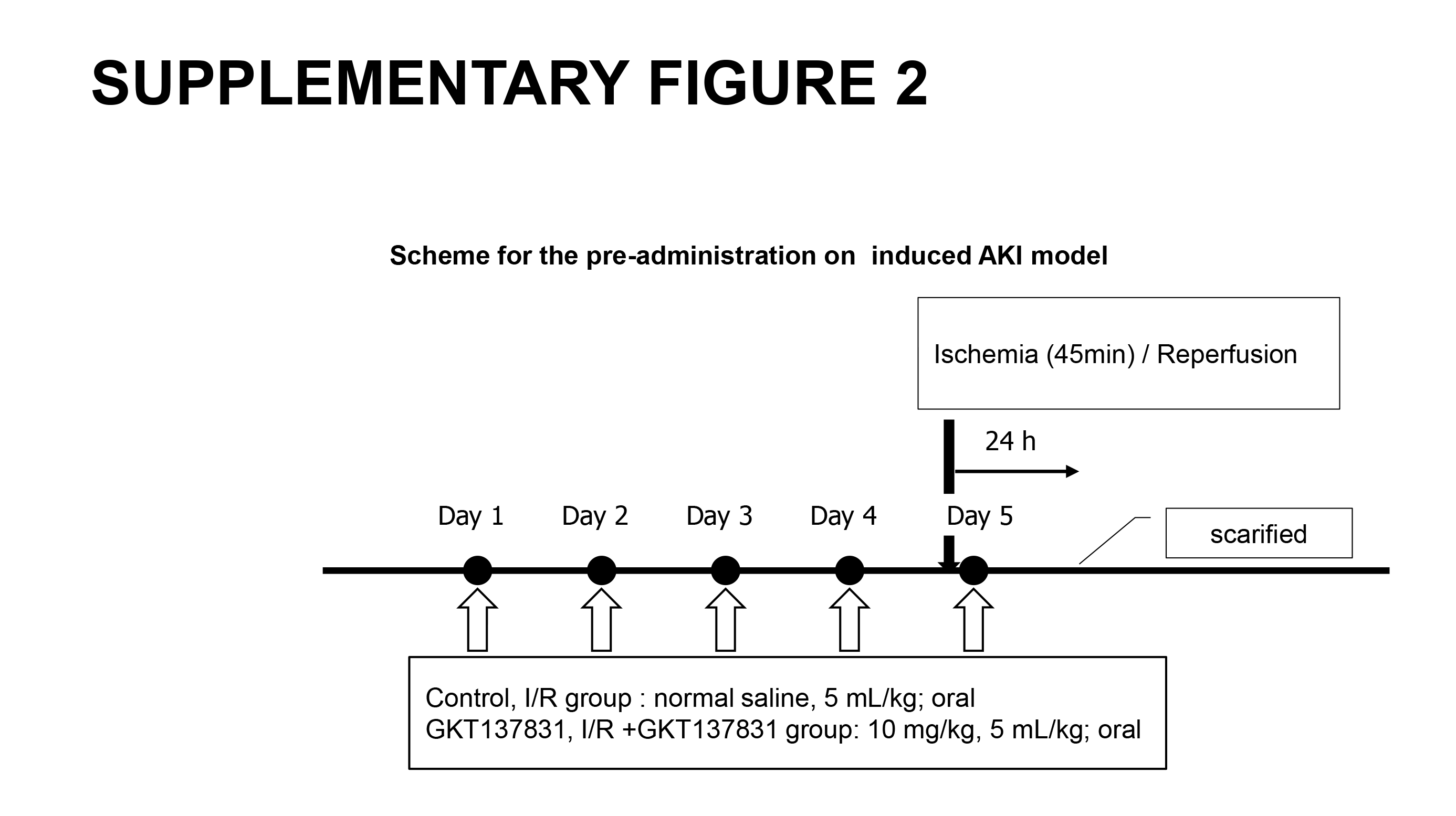

Supplement: S2 Fig — The efficiency of Nox4 knockdown was confirmed by measuring Nox4 mRNA expression and the amount of Nox4 protein by qRT-PCR and western blotting, respectively. (TIF) [file pone.0219483.s002.tif]

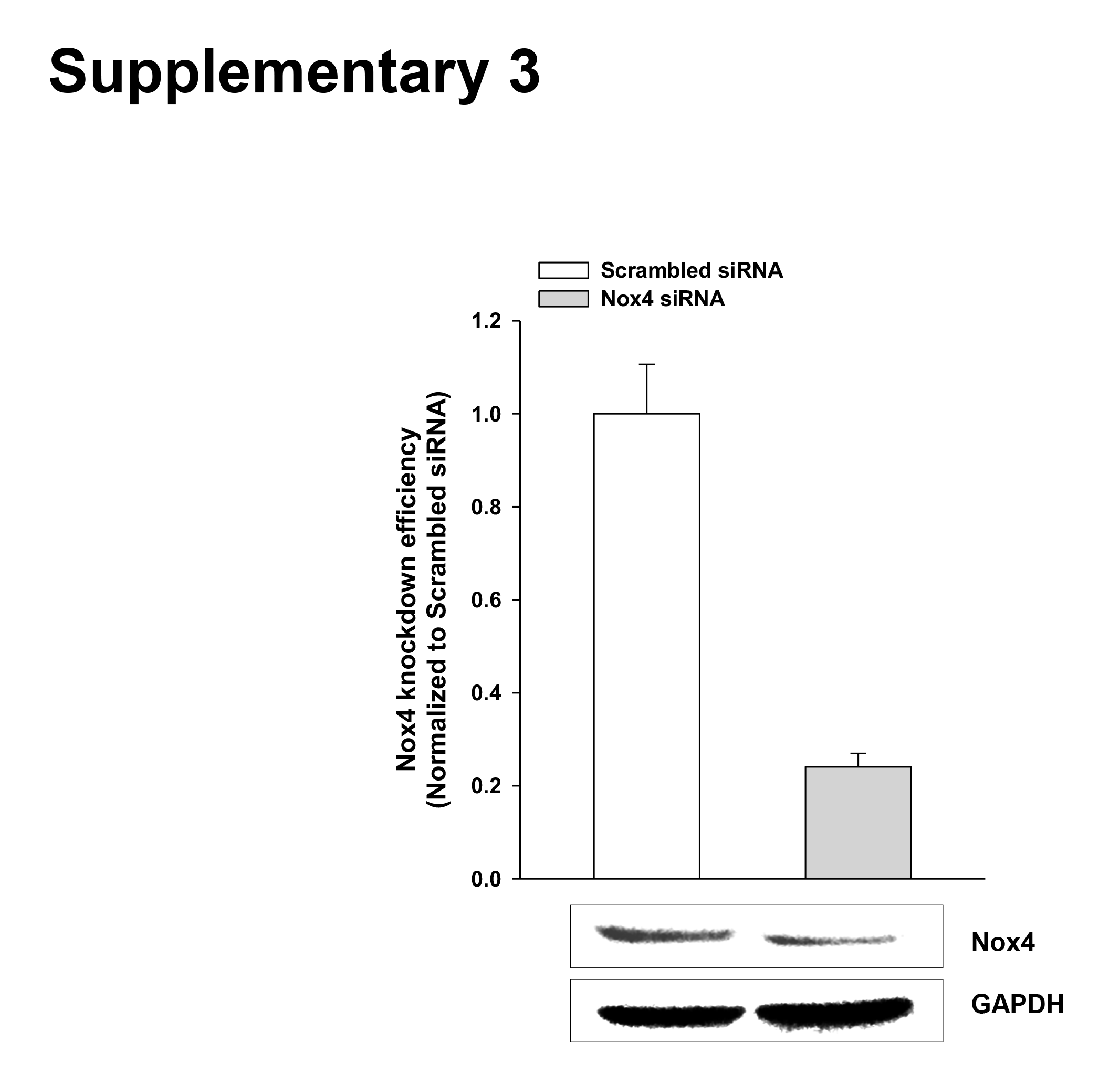

Supplement: S3 Fig — Scheme for the pretreatment of GKT137831 on ischemic reperfusion injury model GKT137831 (10mg/kg) or normal saline (5ml/kg) was administered orally once a day during four days prior to the ischemic injury (45min) and once after ischemic injury. The rats were scarified 24 hours after ischemic injury. (TIF) [file pone.0219483.s003.tif]

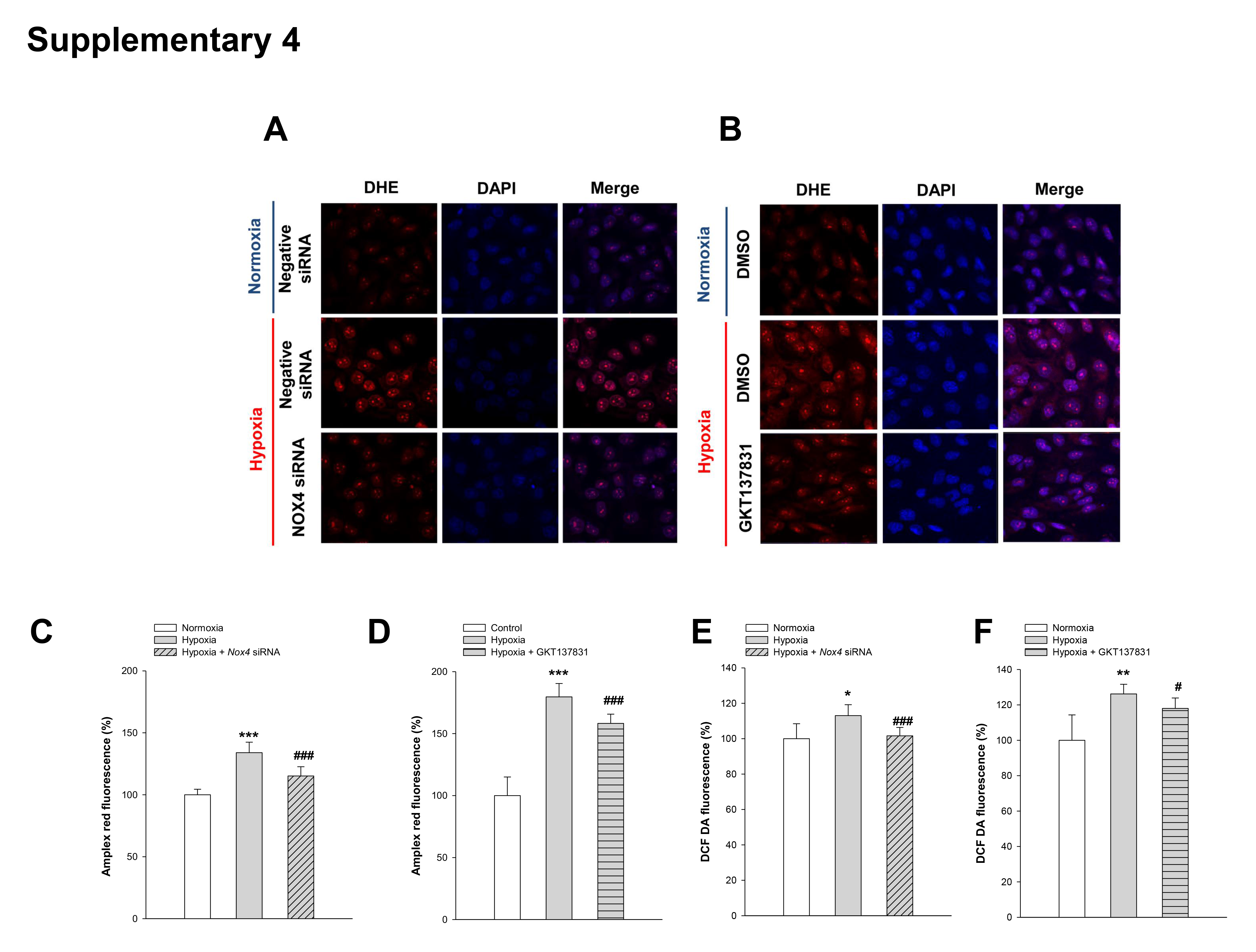

Supplement: S4 Fig — HK-2 cells were exposed to CoCl2. Confocal microscopy images of cells subjected to dihydroethidium (DHE) staining with and without Nox4 knockdown (A) or treated with and without GKT137831 (B). Levels of H2O2, a product of Nox4, were measured by the Amplex red assay with and without Nox4 knockdown (C) or treated with and without GKT137831 (D). Levels of ROS measured by DCF-DA with and without Nox4 knockdown (E) or treated with and without GKT137831 (F). Data represent means ± SD; *p< 0.01,**p < 0.05,***p < 0.001 vs. control and #p < 0.05, ##p < 0.01, ###p<0.001 vs. hypoxia alone. (TIF) [file pone.0219483.s004.tif]
